# Effects of Dexmedetomidine Postconditioning on Myocardial Ischemia/Reperfusion Injury in Diabetic Rats: Role of the PI3K/Akt-Dependent Signaling Pathway

**DOI:** 10.1155/2018/3071959

**Published:** 2018-10-08

**Authors:** Xiangyang Cheng, Jing Hu, Ya Wang, Hongwei Ye, Xiaohong Li, Qin Gao, Zhenghong Li

**Affiliations:** ^1^Department of Anesthesiology, The First Affiliated Hospital of Bengbu Medical College, Bengbu, Anhui 233004, China; ^2^Department of Physiology, Bengbu Medical College, Bengbu, Anhui 233030, China

## Abstract

**Objective:**

The present study was designed to determine whether dexmedetomidine (DEX) exerts cardioprotection against myocardial I/R injury in diabetic hearts and the mechanisms involved.

**Methods:**

A total of 30 diabetic rats induced by high-glucose-fat diet and streptozotocin (STZ) were randomly assigned to five groups: diabetic sham-operated group (DM-S), diabetic I/R group (DM-I/R), diabetic DEX group (DM-D), diabetic DEX + Wort group (DM-DW), and diabetic Wort group (DM-W). Another 12 age-matched male normal SD rats were randomly divided into two groups: sham-operated group (S) and I/R group (I/R). All rats were subjected to 30 min myocardial ischemia followed by 120 min reperfusion except sham groups. Plasmas were collected to measure the malondialdehyde (MDA), creatine kinase isoenzymes (CK-MB), and lactate dehydrogenase (LDH) levels and superoxide dismutase (SOD) activity at the end of reperfusion. Pathologic changes in myocardial tissues were observed by H-E staining. The total and phosphorylated form of Akt and GSK-3*β* protein expressions were measured by western blot. The ratio of Bcl-2/Bax at mRNA level was detected by reverse transcription-polymerase chain reaction (RT-PCR).

**Results:**

DEX significantly reduced plasma CK-MB, MDA concentration, and LDH level and increased SOD activity caused by I/R. The phosphorylation of Akt and GSK-3*β* was increased, Bcl-2 mRNA and the Bcl-2/Bax ratio was increased, and Bax mRNA was decreased in the DEX group as compared to the I/R group, while posttreatment with Wort attenuated the effects induced by DEX.

**Conclusion:**

The results of this study suggest that DEX postconditioning may increase the phosphorylation of GSK-3*β* by activating the PI3K/Akt signaling pathway and may inhibit apoptosis and oxidative stress of the myocardium, thus exerting protective effects in diabetic rat hearts suffering from I/R injury.

## 1. Introduction

The incidence of type-2 diabetes mellitus (T2DM) is on the increase worldwide which seriously threatens the health of the patients. Diabetes is a common metabolic disorder characterised by hyperglycemia, hyperinsulinemia, and insulin resistance leading to pathological changes in multiple organs, among which heart disease is the most common [1].

Myocardial ischemia/reperfusion (I/R), known for restoration of blood supply to ischemic areas, causes further injury such as arrhythmias, mitochondrial dysfunction, intracellular calcium overload, oxidative stress, cell apoptosis, and even cell death [2, 3].

The risk of heart failure and fatalities after myocardial I/R in the diabetic is 2-3 times higher than the nondiabetic [4, 5]. Strategies such as ischemic postconditioning aiming to alleviate myocardial I/R injury have been validated both in animal models as well as clinical trials [6–8]. Comparatively, pharmacological postconditioning has been widely used due to its more implementable and less invasive nature [9]. Postischemic treatment with anesthetic agents, such as remifentanil, sevoflurane, and morphine, has been suggested to induce cardioprotective effects in normal rats [10–12], while additional studies have shown that the cardioprotection of ischemic postconditioning and some anesthetic treatment may be blocked or abrogated in pathological conditions such as diabetes, hypercholesterolemia, and hyperglycemia [10–13]. Therefore, a novel pharmacological agent treatment aiming to improve myocardial functional recovery, reduce myocardial apoptosis, and protect ischemic myocardium in diabetes with I/R injury needs more attention. Dexmedetomidine (DEX), known as a highly selective *α*_2_-adrenergic receptor agonist, has been widely used for sedation and analgesia in anaesthesia as well as in the intensive care unit. Our previous study [14] along with other reports [3, 15] has shown that DEX administration improves cardiac function, suppresses apoptosis, and reduces the infarct area and oxidative stress in nondiabetic hearts suffering from I/R. And the cardioprotection of DEX may be related with activation of the phosphatidylinositol-3 kinase/protein kinase B (PI3K/Akt) signaling pathway [3, 14]. The PI3K/Akt signaling pathway has been confirmed to exert cardioprotection against I/R by regulating cell proliferation, suppressing apoptosis, and reducing the release of oxygen free radicals [2], while some reports suggested that the PI3K/Akt signaling pathway was altered in the diabetic state, resulting in the abrogation of protective effects of pharmacological agents as well as the ischemic postconditioning method induced in normal rats' hearts [4]. However, whether DEX-induced cardioprotection against I/R injury remains in diabetes is still not clear. GSK-3*β*, an important intracellular protein known as downstream of the Akt pathway, also linked with oxidative stress and cell apoptosis and exert a key role in diabetes mellitus [16, 17]. It has been confirmed that the inhibition activity of GSK-3*β* exerts protective effects on the brain, heart, and kidney in both healthy and diabetic conditions [6, 12, 17] while amounts of reports showed that GSK-3*β* is activated in diabetes due to the impairment of its upstream pathways, thus affecting the protective effect of postconditioning methods as well as pharmacological agents [11–13]. However, it has not yet been reported whether DEX postconditioning could attenuate I/R in diabetic rats and the underlying mechanisms. Therefore, the study sets out with the aim of assessing the cardioprotective effect of DEX and then to determine whether activation of the PI3K/Akt pathway as well as inhibition of downstream protein glycogen synthasekinase-3*β* (GSK-3*β*) is involved.

## 2. Materials and Methods

### 2.1. Animals

Male Sprague-Dawley rats (130–150 g) were supplied by the Animal Center of Bengbu Medical College, Anhui (license no. SCXK (Hu) 2013-0006). All rats housing at 22–26°C and 50–70% humidity with 12 h light/dark cycle were allowed free access to food and water. Experimental procedures were conducted according to the guidelines established by the Committee of Bengbu Medical College for laboratory animal use and care.

### 2.2. Chemicals and Reagents

Streptozotocin (STZ) and wortmannin (Wort) were purchased from Sigma-Aldrich (St. Louis, MO, USA); dexmedetomidine (DEX) was purchased from Hengrui Pharmaceutical Co. (Jiangsu, China). Creatine kinase isoenzymes (CK-MB), lactate dehydrogenase (LDH), malondialdehyde (MDA), and superoxide dismutase (SOD) assay kits were obtained from Jiancheng Institute of Biotechnology (Nanjing, China). Primary antibodies obtained from Cell Signaling Technology Inc. (Danvers, MA, USA) were as follows: GSK-3*β* (BF0695), p-GSK-3*β* (AF2016), Akt (9272S), p-Akt (4060S), and *β*-actin (8H10D10). Rabbit anti-mouse and goat anti-mouse secondary antibodies were obtained from Boston Co. Ltd. (Wuhan, China). Bcl-2, Bax, and *β*-actin primers were obtained from Sangon Biotech Co. Ltd. (Shanghai, China) (Table 1).

### 2.3. Induction of Diabetes

After acclimating to laboratory condition for one week, rats were fed high-fat diet (consisting of 74.5% basic diet, 10% sugar, 10% fat, 5% egg, and 0.5% cholesterol). After 4 weeks of dietary intervention, rats underwent fasting for 12 h then were treated with an injection of 1% STZ 35 mg/kg (dissolved in 0.1 mol/l citrate buffer, pH 4.5) intraperitoneally for diabetes induction, while normal rats were injected with a citrate buffer vehicle. Seventy-two hours later, blood samples from the tail vein were collected to measure fasting blood glucose (FBG) using a portable glucometer (Accu-Chek, Roche, Germany). Only rats with FBGs higher than 16.7 mmol/l along with symptoms including polydipsia, polyphagia, and polyuria were considered diabetic. Body weight (BW) and FBG were monitored once a week. After 4 weeks of STZ injection, rats were exposed to ischemia/reperfusion. Similarly, normal rats were kept for 8 weeks until further study [18].

### 2.4. Experimental Design

Diabetic rats were randomly divided into the following five groups (*n* = 6): diabetic sham-operated group (DM-S), diabetic I/R group (DM-I/R), diabetic DEX group (DM-D), diabetic DEX + Wort group (DM-DW), and diabetic Wort group (DM-W). Another age-matched male normal SD rats were randomly assigned into two groups (*n* = 6): sham-operated group (S) and I/R group (I/R). The sham-operated group rats were treated with a left anterior descending coronary artery (LAD) thread without ligation for 150 min. Rats in the I/R group underwent LAD ligation for 30 min followed by 120 min of reperfusion. DEX group rats were given an intravenous injection with DEX at a dose of 10 *μ*g/kg [19] 5 min before reperfusion and then subjected to 120 min of reperfusion; diabetic DEX + Wort group (DM-DW) and diabetic Wort group (DM-W) rats were given Wort intravenously at a dose of 15 *μ*g/kg [14] 15 min prior to 120 min of reperfusion with or without DEX posttreatment.

### 2.5. Induction of Ischemia/Reperfusion

All rats were anesthetized with intraperitoneal injections of 4% chloral hydrate (0.9 ml/100 g body weight). Electrocardiogram (ECG) information were collected continuously. Following tracheal intubation, mechanical ventilation was started with room air, using a tidal volume of 2 ml/100 g body weight at 65–75 breaths/min. The chest was gently opened between the third and fourth rib along the left sternal; after separating the pericardium, the heart was exposed. Then a single 5-0 Prolene suture was threaded through the LAD. Rats were subjected to 30 min of regional ischemia by ligating the artery, and then the ligature was loosened for 120 min as reperfusion, while sham-operated group rats underwent the same progress except ligating the artery. The rats' body temperature was kept at 37°C by a heating pad during surgery [10].

### 2.6. Measurement of H/B

At the end of reperfusion, hearts were immediately excised and washed with precooled normal saline. Then heart weights were measured. The heart weight to body weight ratio was calculated as a cardiac index (H/B, mg/g).

### 2.7. Measurement of Plasma TG, TC, CK-MB, LDH, SOD, and MDA Levels

Blood samples from an abdominal artery were collected at 120 min of reperfusion. After centrifugation (3000 rpm, 15 min, 4°C), the plasma samples were collected and stored at −80°C until assayed. Triglycerides (TG) and total cholesterol (TC) levels were determined by an automatic biochemistry analyzer. CK-MB concentration was determined by the ELISA kit according to the manufacturer instructions. MDA concentration and LDH and SOD activities were measured by colorimetric assay kits in accordance with the manufacturer protocols.

### 2.8. Measurement of Akt, p-Akt, GSK-3*β*, and p-GSK-3*β* by Western Blot

Myocardium tissues (100 mg) were collected and homogenized in a lysis buffer then centrifuged at 12,000 rpm for 15 min at 4°C. The protein concentration was determined by the BCA Protein Assay kit. The total protein (40 g) of each sample was separated by 12% SDS-polyacrylamide gel electrophoresis and transferred to a PVDF membrane (Millipore Co., Billerica, MA) at 200 mA for 2 h. Thereafter, the membranes were blocked with 5% nonfat milk or 5% bovine serum albumin in Tris-buffered saline containing 0.1% Tween-20 (TBST) for 2 h at room temperature and then incubated with the corresponding primary antibody, including rabbit Akt antibody (1 : 1000), rabbit p-Akt antibody (1 : 1000), mouse GSK-3*β* antibody (1 : 500), rabbit p-Akt GSK-3*β* antibody (1 : 1000), and mouse *β*-actin antibody (1 : 1000) at 4°C overnight. Washed with TBST, membranes were incubated with secondary antibody HRP-linked anti-mouse IgG or HRP-linked anti-rabbit IgG for 1 h. After incubation with the enhanced chemiluminescence (ECL) reagents, the bands were scanned and quantified with ImageJ software.

### 2.9. Measurement of Bax and Bcl-2 mRNA Expressions by RT-PCR

The total RNA (3 g) was extracted with TRIzol (Invitrogen, USA) and reverse-transcribed to cDNA then PCR was performed using a routine method. Densitometry results for Bax and Bcl-2 genes were compared with corresponding *β*-actin levels to account for loading differences.

### 2.10. H-E Staining

At the end of the reperfusion, left ventricular myocardial tissues were harvested for histological examination and fixed in 4% paraformaldehyde; then the myocardial tissue was dehydrated with ethanol and embedded in paraffin. Thereafter, the myocardial tissues were cut into serial sections (5 *μ*m) and stained with hematoxylin and eosin (H-E) dye.

### 2.11. Statistical Analysis

All values were expressed as mean ± SD. Comparisons between the two groups were analyzed by Student's *t*-test. Multiple comparisons were performed using the one-way analysis of variance (ANOVA) followed by Student-Newman-Keuls (SNK). *P* < 0.05 was regarded as statistically significant.

## 3. Results

### 3.1. General Characteristics at Termination

There was no difference in body weight among rats in each group at the beginning of the experiment. However, 4 weeks after STZ injection, body weight in diabetic rats was significantly lower and H/B were higher than normal rats (*P* < 0.05). Besides, increased water intake and food consumption were observed in diabetic rats. The levels of FBG, TG, and TC in diabetic rats were increased compared to normal rats (*P* < 0.05) (Table 2).

### 3.2. Alterations to the Plasma CK-MB, LDH, SOD, and MDA Levels in Each Group

**Figure 1:**
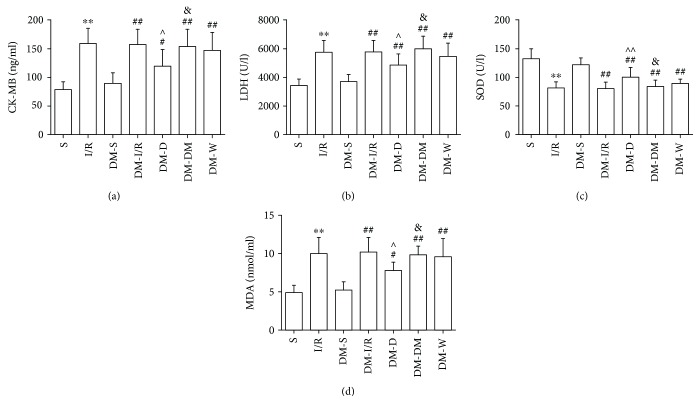
The levels of CK-MB (a), LDH (b), SOD (c), and MDA (d) in the serum of rats from each group (mean ± SD, *n* = 6). ^∗∗^*P* < 0.01 compared with S; ^#^*P* < 0.05; ^##^*P* < 0.01 compared with DM-S; ^∧^*P* < 0.05; ^∧∧^*P* < 0.01 compared with DM-I/R; ^&^*P* < 0.05 compared with DM-D; S: sham-operated group; I/R: ischemia/reperfusion group; DM-S: diabetic sham-operated group; DM-I/R: diabetic ischemia/reperfusion group; DM-D: diabetic dexmedetomidine group; DM-DW: diabetic dexmedetomidine + wortmannin group; DM-W: diabetic wortmannin group.

Compared with the S and DM-S groups, CK-MB, LDH, and MDA levels were increased; SOD activity was decreased in all of the other groups. Compared with I/R and DM-I/R groups, CK-MB, LDH, and MDA levels were decreased in the DM-D group and SOD activity was increased. Compared with the DM-D group, CK-MB, LDH, and MDA levels were significantly increased in the DM-DW group and SOD activity was decreased in the DM-DW group (Figure 1).

### 3.3. Changes of Akt, p-Akt, GSK-3*β*, and p-GSK-3*β* Protein Levels in the Myocardial Tissue

**Figure 2:**
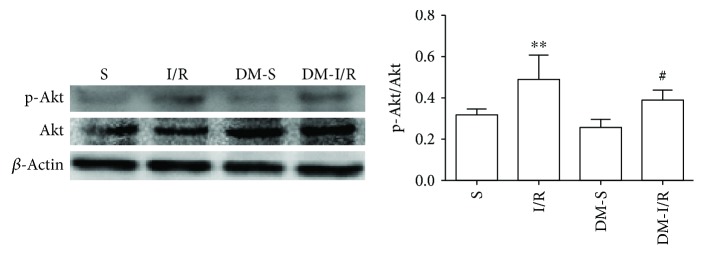
Expression levels of p-Akt in heart tissue (mean ± SD, *n* = 4) ^∗∗^*P* < 0.01 compared with S; ^#^*P* < 0.05 compared with DM-S.

**Figure 3:**
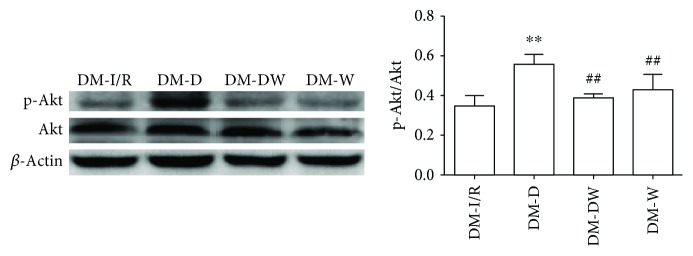
Expression levels of p-Akt in heart tissue (mean ± SD, *n* = 4) ^∗∗^*P* < 0.01 compared with DM-I/R; ^##^*P* < 0.01 compared with DM-D.

**Figure 4:**
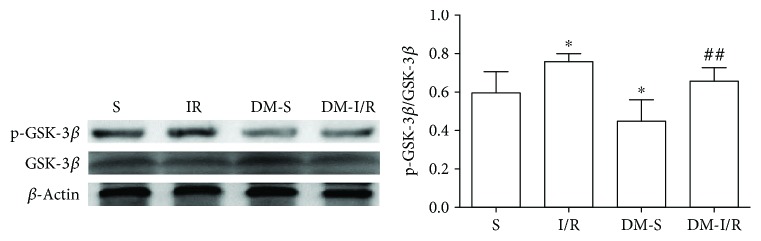
Expression levels of p-GSK-3*β* (Ser9) in heart tissue (mean ± SD, *n* = 4). ^∗^*P* < 0.05 compared with S; ^##^*P* < 0.01 compared with DM-S.

**Figure 5:**
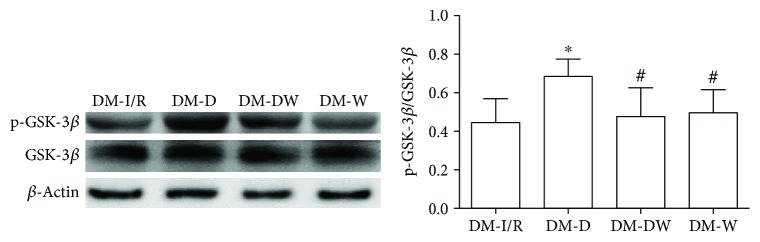
Expression levels of p-GSK-3*β* (Ser9) in heart tissue (mean ± SD, *n* = 4). ^∗^*P* < 0.05 compared with DM-I/R; ^#^*P* < 0.05 compared with DM-D.

Compared with S and DM-S groups, the p-Akt/Akt ratio and p-GSK-3*β*/GSK-3*β* ratio were increased when suffered from I/R injury (*P* < 0.05) (Figures 2–5). The p-Akt/Akt ratio was not significantly different in the S group and DM-S group (*P* < 0.05), while the p-GSK-3*β*/GSK-3*β* ratio in the DM-S group was lower than the S group (*P* < 0.05). Compared with the DM-I/R group, the p-Akt/Akt ratio and p-GSK-3*β*/GSK-3*β* ratio were increased in the DM-D group, respectively (*P* < 0.05). Compared with the DM-D group, p-Akt/Akt ratio and p-GSK-3*β*/GSK-3*β* ratio in the DM-DW group were decreased (*P* < 0.05).

### 3.4. Changes of Bcl-2 and Bax mRNA Levels in the Myocardial Tissue

**Figure 6:**
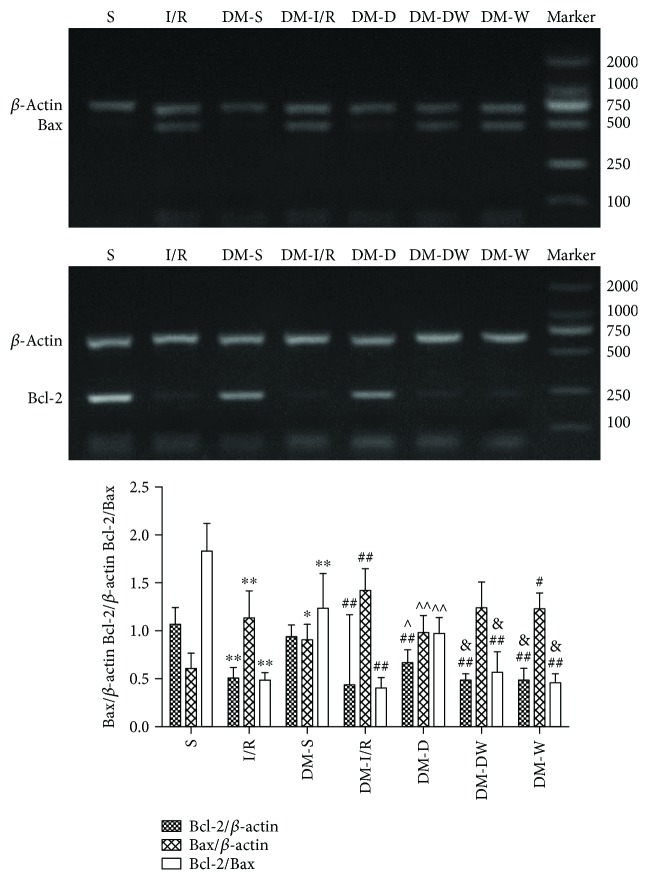
The expressions of Bcl-2 and Bax mRNA in heart tissue (mean ± SD, *n* = 4). ^∗^*P* < 0.05; ^∗∗^*P* < 0.01 compared with S; ^#^*P* < 0.05; ^##^*P* < 0.01 compared with DM-S; ^∧^*P* < 0.05; ^∧∧^*P* < 0.01 compared with DM-I/R; ^&^*P* < 0.05 compared with DM-D.

The level of gene Bcl-2 was similar between S and DM-S groups, while the Bax mRNA level was increased and the Bcl-2/Bax ratio was decreased in the DM-S group compared with the S group (*P* < 0.05). Compared with the DM-I/R group, Bax mRNA was decreased and the Bcl-2 mRNA and Bcl-2/Bax ratio were increased in the DM-D group (*P* < 0.05). Compared with the DM-D group, Bcl-2 mRNA and Bcl-2/Bax ratio were decreased in the DM-DW group (*P* < 0.05) (Figure 6).

### 3.5. The Pathologic Changes in the Myocardial Tissue

**Figure 7:**
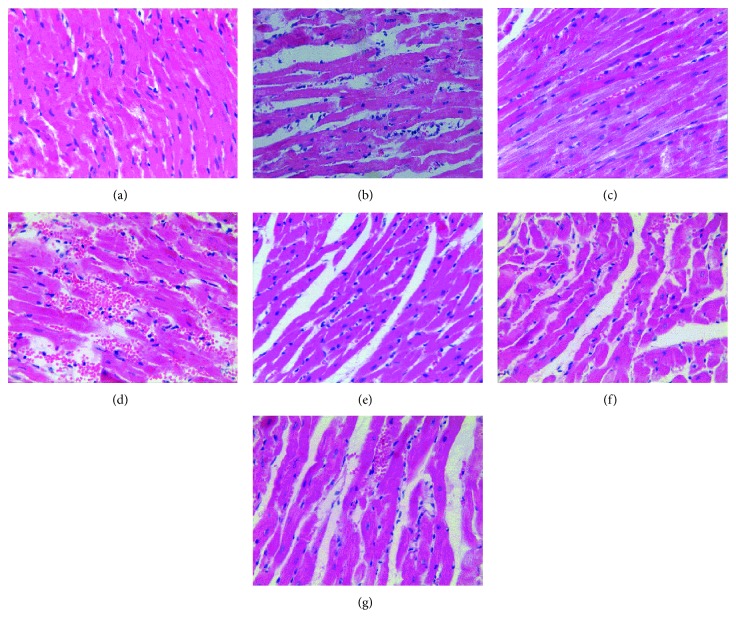
The result of hematoxylin and eosin (H-E) staining cardiomyocytes (original magnification: ×400). (a) S group, (b) I/R group, (c) DM-S group, (d) DM-I/R group, (e) DM-D group, (f) DM-DW group, and (g) DM-W group.

The myocardial cells were arranged regularly; the envelope was intact; and no inflammatory cells or red blood cells were observed in the cytoplasm in the S group, while inflammatory cells were observed in the DM-S group. Myocardial fiber disarrangement, neutrophil infiltration, myofibrillar discontinuation, and enlarged intercellular space were observed in I/R, DM-I/R, DM-DW, and DM-W groups. DEX posttreatment ameliorated those histological changes in the hearts (Figure 7).

## 4. Discussion

It has been reported that DEX had protective effects in the hippocampus [20], spinal cord [21], heart [14], liver [22], and kidney [19] in normal rats through the antioxidative and anti-inflammatory effects. The protective effect of DEX was also observed in diabetic rats [23, 24]. The type 2 diabetic rat model, exposed to I/R, were induced by high-glucose-fat diet with low-dose STZ in this study. Body weight, FBG, plasma TG, and TC of diabetic rats were increased compared to normal rats; meanwhile, heart weight in these rats was decreased in the 4th week after STZ injection, indicating that diabetes may cause lipid metabolism disorder and cardiac hypertrophy.

During I/R, the myocardial membrane integrity was lost, and the myocardial enzymes such as CK-MB and LDH were released into the plasma. Therefore, plasma CK-MB and LDH levels were determined as an indicator of myocardial tissue damage. Following 30 min ischemia and 120 min reperfusion, the levels of plasma CK-MB and LDH were increased in all of the other groups than S and DM-S groups, suggesting the successful establishment of the I/R injury model. DEX administration reduced levels of plasma CK-MB and LDH in the DM-I/R group, indicating the protective effects of DEX in diabetic condition. Meanwhile, plasma CK-MB and LDH levels in the DM-DW group were increased compared to the DM-D group, suggesting that cardioprotection of DEX were suppressed by Wort. However, the aforementioned index in the DM-S group was similar to the S group, and no significant difference was observed between DM-S and S groups, indicating that a 4-week diabetic state may not significantly influence cardiac function, which was consistent with a previous study [25], while Mokhtari et al. reported that STZ-induced diabetes at the 10th week significantly increases the level of cTnI and exacerbates the myocardial injury [26]. Besides, prior studies have noted that a diabetic heart is more sensitive to ischemic injury [26, 27], while Aasum et al. [28] have reported that the in vitro diabetic rat heart was less sensitive to myocardial I/R injury 6 weeks after STZ administration, while at 12th week, cardiac function was significantly impaired. In our study, no significant difference was observed between I/R and DM-I/R groups. The controversial results may be associated with a difference of experimental design and diabetic duration.

Oxidative stress is involved in the occurrence and development of diabetes mellitus [29]. The oxidative stress of diabetes can aggravate myocardial I/R injury. Studies have shown that the myocardial oxidative stress indexes such as SOD and CAT were decreased after I/R in the diabetic myocardium [30], and myocardial I/R injury exert excessive amounts of reactive oxygen species and exaggerates development of myocardial injury [31]. As expected, MDA level was increased, and SOD activity was increased in all the other groups compared to S and DM-S groups. DEX postconditioning decreased the MDA level and increased SOD activity in rats suffering I/R, while the presence of Wort reversed the effect. DEX postconditioning can attenuate oxidative stress in diabetes, which was consistent with a previous report [24]. These data taken together showed the antioxidant stress effects of DEX in diabetic rat hearts.

Previous studies had demonstrated that the Reperfusion Injury Salvage Kinase (RISK) pathway including Phosphatidylinositol 3 kinase/protein kinase B (PI3K/Akt) and extracellular signal-regulated kinase 1/2 (ERK1/2) were activated to possess protective effects at the onset of myocardial reperfusion followed by ischemia. The PI3K/Akt signaling pathway plays a vital role in cell survival, growth and proliferation, and resistance to I/R injury [32]. It has been reported that DEX exhibited protective properties in the rat's hippocampus through the PI3K/Akt and ERK1/2 signaling pathways by reducing cerebral infarct volume and improving the neurological deficit score [20]. We previously discovered that DEX exerts cardioprotective effects against myocardial I/R injury in normal rats via the PI3K/Akt/GSK-3*β* pathway [13]; herein, we explored the potential cardioprotective effects in diabetic rats. In the present study, we found out that p-Akt and p-GSK-3*β* were upregulated by DEX in the diabetic I/R rat heart. However, the enhancement of p-Akt and p-GSK-3*β* induced by DEX was depressed by treatment with wortmannin, a PI3K/Akt inhibitor. The phosphorylation of Akt in the DM-S group was not different from the S group, which was not consistent with the Wang et al. study, which reported that the signaling pathway was impaired in the diabetic myocardium, with a lower level of basal Akt phosphorylation than normal rats [27]. The phosphorylation of GSK-3*β* was decreased in the DM-S group compared with the S group, suggesting that p-GSK-3*β* was inactivated in the diabetic state, which was confirmed by a prior study [25]. Mokhtari et al. [26] confirmed that at the 10th week of diabetes induction, phosphorylation of GSK-3*β* in the DM-I/R group was significantly less than the I/R group, suggesting that diabetes impairs the intracellular signaling pathways and increases cardiac injury as compared to normal hearts; however, no significant difference was seen between I/R and DM-I/R groups in our study. GSK-3*β*, known as a proapoptotic kinase, is the downstream target of the PI3K/Akt and plays a key role in pharmacological cardioprotection against I/R injury in a diabetic. Previous studies have reported that inactivation of GSK-3*β* exerts protection in diabetic rat hearts as well as distant organs including the brain caused by myocardial I/R injury [6]. Regulated by activation of Akt, by which GSK-3*β* is phosphorylated at Ser9 and is thereby inactivated. Phosphorylation of GSK-3*β*, which binds to the mPTP subunit adenine nucleotide transporter (ANT), increases mPTP open threshold, thereby inhibiting mPTP opening and protecting the integrity of mitochondrial membrane, which is considered to be one of the decisive factors to inhibit mPTP opening [33]. Studies confirmed that mechanical interventions ischemic postconditioning and preconditioning or postconditioning with sevoflurane, propofol, and other pharmacological agents could increase the level of mitochondrial p-GSK-3*β*, increase the threshold of mPTP opening, and protect the myocardium [11–13, 26, 33, 34]. The results suggested that DEX exerts cardioprotective effects against I/R injury in diabetic rats by activating the PI3K/Akt pathway and phosphorylating downstream protein GSK-3*β*. We speculated that when GSK-3*β* is phosphorylated at Ser9 by treatment with DEX, the opening of the mPTP is suppressed and then the improve cell survive. However, the hypothesis needs to be clarified in future study.

PI3K dependent Akt activation and its phosphorylation preserve mitochondrial integrity and protect the cardiac cells by attenuating apoptosis. When Akt is activated, it may cause phosphorylation of Bad or Bax residues, regulating the activity of Bcl-2, thus exerting an antiapoptotic effect during myocardial ischemia [35]. The GSK-3*β* inactive form, increase the mPTP opening threshold, followed by inhibiting proapoptotic signals cytochrome C release, suppressing the caspases activation and eventually leading cell death [26]. Apoptosis is one of the mechanisms in I/R injury and involved in cardiovascular complications of diabetes mellitus [26, 36]. The activation of caspase-3 and the release of mitochondrial cytochrome C caused by hyperglycemia lead to myocardial cell apoptosis [10, 37]. Bcl-2 gene family is known to regulate the mitochondrial changes including modulation the permeability of the mitochondrial membrane and release of cytochrome C during apoptosis [38]. The balance of the antiapoptotic Bcl-2 and the proapoptotic Bax genes expression level serves a crucial function in regulating myocardial apoptotic cell death. The ratio of Bcl-2/Bax indicates the extent of apoptosis. In our study, we found out that the Bax mRNA expression was higher in the DM-S group, while Bcl-2 mRNA expression was similar between DM-S and S groups, and the Bcl-2/Bax ratio was lower in the DM-S group, suggesting that cell apoptosis was induced by diabetes. Kim et al. [10] reported that in the T1M rat model, 3 weeks after STZ administration, Bax and cytochrome C protein expressions were higher in the diabetic heart, while the Bcl-2 protein expression was similar between normal and diabetic rats; the results were in accordance with ours. Besides, DEX post-conditioning significantly increased the Bcl-2/Bax ratio in diabetic rat hearts suffering I/R injury, suggesting that the decrease in myocardial cells apoptosis may be associated with DEX. To investigate whether the PI3K/Akt/GSK-3*β* signaling pathway was related to the antiapoptotic effect of DEX, the PI3K inhibitor wortmannin (Wort) was adopted in this study. The findings showed that the ratio of Bcl-2/Bax mRNA was decreased as the presence of Wort in diabetic rat hearts, suggesting that the inhibition of cardiomyocyte apoptosis by DEX during I/R is partial via the PI3K/Akt/GSK-3*β* signaling pathway, which were reinforced by the study of Zhang WP et al. [39]. Studies have demonstrated DEX attenuates neuroapoptosis via the PI3K/Akt pathway [40, 41], indicating that the PI3K/Akt pathway was involved in the antiapoptotic effect of DEX.

Nevertheless, there were some limitations in the present study. Only one dose of DEX was adopted in the study; we did not investigate the dose-effect of DEX postconditioning on myocardial I/R. Besides, a single factor is insufficient to elucidate the variance in the effects of DEX in diabetes and normal rats as a result of differences in experimental design and animal models. Rats in the S group show more antiapoptotic than the DM-S group, while other indicators such as the oxidative stress index and myocardial enzyme spectrum index aforementioned were not significantly different between the two groups, and whether the PI3K/Akt/GSK-3*β* signaling pathway was impaired in diabetes needs further study.

Taken together, it seems that DEX postconditioning may increase the phosphorylation of GSK-3*β* by activating the PI3K/Akt signaling pathway and may inhibit apoptosis and oxidative stress of the myocardium, thus exerting protective effects in diabetic rat hearts suffering from I/R injury.

## Figures and Tables

**Table 1:** Reverse transcription-polymerase chain reaction primers for Bax, Bcl-2 and β-actin.

Gene	Primer	Sequence	Product(bp)
Bax	Forward	5′-GGA TCG AGC AGA GAG GAT GG-3′	464
	Reverse	5′-TGG TGA GTG AGG CAG TGA GG-3′	
Bcl-2	Forward	5′-CTG GTG GAC AAC ATC GCT CTG-3′	227
	Reverse	5'-GGT CTG CTG ACC TCA CTT GTG-3'	
β-actin	Forward	5′-GAT GGT GGG TAT GGG TCA GAA GGA C-3′	630
	Reverse	5′-GCT CAT TGC CGA TAG TGA TGA CT-3′	

**Table 2:** Characteristics of rats in different groups.

Parameters	Normal	DM
BW(g)	372±26	318±50^∗∗^
HW(g)	1.19±0.06	1.03±0.14^∗∗^
H/B(%)	0.32±0.03	0.33±0.03
FBG(mmol/L)	5.94±0.91	25.28±3.86^∗∗^
TC (mmol/L)	1.78±0.31	3.69 ± 0.75^∗∗^
TG (mmol/L)	0.79±0.11	3.02±0.87^∗∗^

Values are means ± SD (*n* = 10). ^∗∗^*P* < 0.01 compared with Normal.

## Data Availability

The data used to support the findings of this study are available from the corresponding author upon request.
